# The adhesion GPCR Adgrg6 (Gpr126): Insights from the zebrafish model

**DOI:** 10.1002/dvg.23417

**Published:** 2021-03-18

**Authors:** Sarah Baxendale, Anzar Asad, Nahal O. Shahidan, Giselle R. Wiggin, Tanya T. Whitfield

**Affiliations:** ^1^ Department of Biomedical Science, Bateson Centre and Neuroscience Institute University of Sheffield Sheffield UK; ^2^ Sosei Heptares, Steinmetz Building Granta Park Cambridge UK

**Keywords:** Adgrg6, aGPCR, drug screening, Gpr126, heart, inner ear, myelination, skeleton, zebrafish

## Abstract

Adhesion GPCRs are important regulators of conserved developmental processes and represent an untapped pool of potential targets for drug discovery. The adhesion GPCR Adgrg6 (Gpr126) has critical developmental roles in Schwann cell maturation and inner ear morphogenesis in the zebrafish embryo. Mutations in the human *ADGRG6* gene can result in severe deficits in peripheral myelination, and variants have been associated with many other disease conditions. Here, we review work on the zebrafish Adgrg6 signaling pathway and its potential as a disease model. Recent advances have been made in the analysis of the structure of the Adgrg6 receptor, demonstrating alternative structural conformations and the presence of a conserved calcium‐binding site within the CUB domain of the extracellular region that is critical for receptor function. Homozygous zebrafish *adgrg6* hypomorphic mutants have been used successfully as a whole‐animal screening platform, identifying candidate molecules that can influence signaling activity and rescue mutant phenotypes. These compounds offer promise for further development as small molecule modulators of Adgrg6 pathway activity.

## 1 INTRODUCTION

**FIGURE 1:**
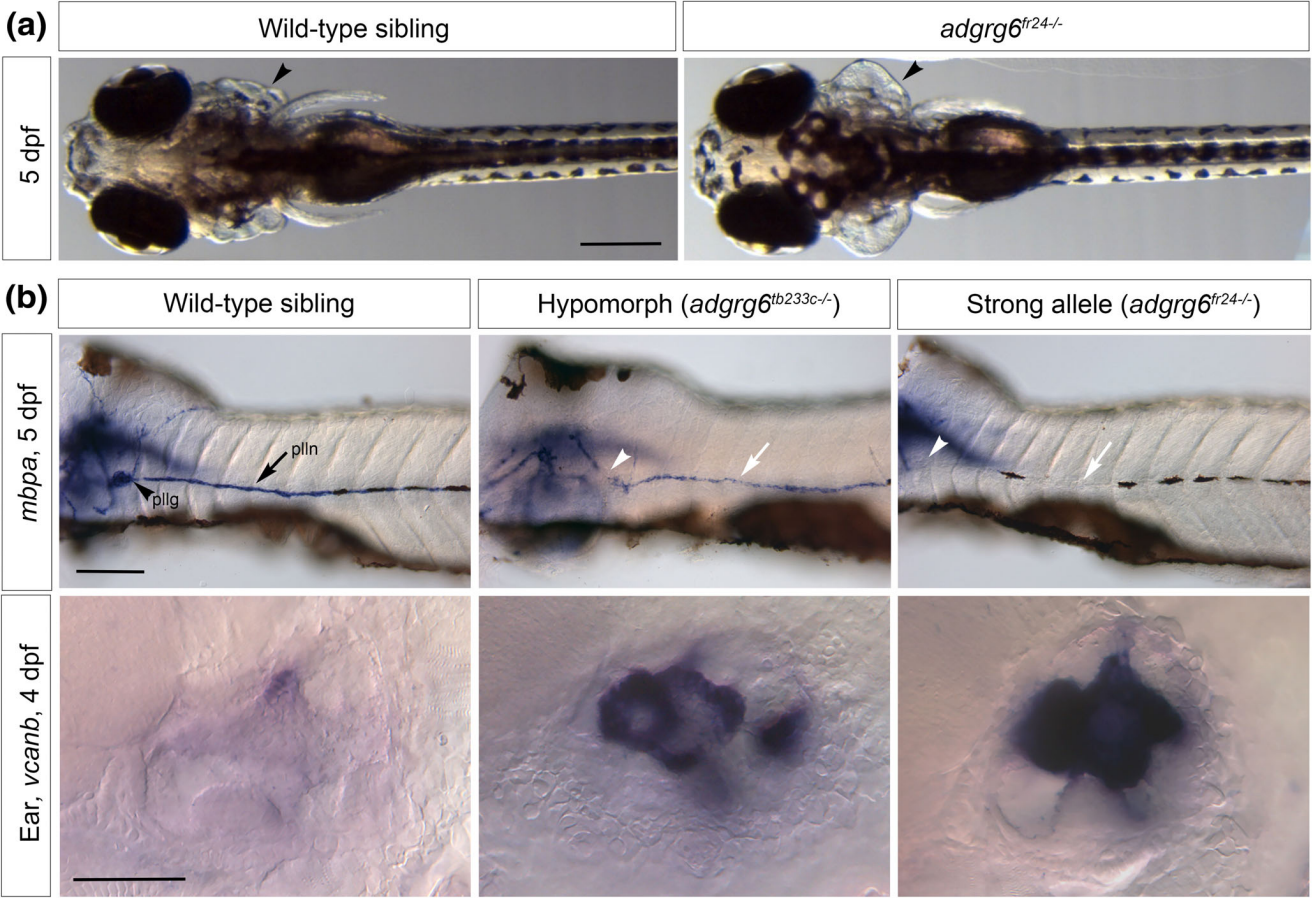
Otic and myelination phenotypes in zebrafish *adgrg6* mutants. (a) Phenotypically wild‐type sibling (left panel, ventral view) and homozygous *adgrg6* ^*fr24−/−*^ mutant (right panel, dorsal view), showing swollen otic vesicles (arrowhead). Anterior to the left. Note that head and eye size, pectoral fins, pigmentation, and swim bladder inflation are all normal in the mutant. Scale bar, 200 μm. Images reproduced from Geng et al., 2013. (b) In situ hybridization to *myelin basic protein a* (*mbpa*) transcripts in the trunk (top row) and to *versican b* (*vcanb*) in the ear (bottom row). Lateral views; anterior to the left. Top row: expression of *mbpa* in Schwann cells of the posterior lateral line ganglion (pllg, arrowhead) and posterior lateral line nerve (plln, arrow) is reduced in the hypomorphic *tb233c* allele, and lost altogether in the strong *fr24* allele. The blurred stain to the left in each image is expression in the central nervous system, which is unaffected in the mutants. Scale bar, 100 μm. Images reproduced from Geng et al., 2013. Bottom row: strong expression of *vcanb* in the ear persists abnormally in *adgrg6* mutants. Scale bar, 50 μm. dpf, days post fertilization. Images reproduced from Diamantopoulou et al., 2019

Adhesion G‐protein‐coupled receptors (aGPCRs) are the second largest group of GPCRs, and are regulators of a wide range of developmental and physiological processes. Like other GPCRs, aGPCRs have a 7‐transmembrane domain, but are specifically characterized by their very large extracellular region (ECR), which includes a GPCR autoproteolysis‐inducing (GAIN) domain (see Bondarev et al., 2020; Langenhan, 2019; Morgan et al., 2019; Vizurraga, Adhikari, Yeung, Yu, & Tall, 2020 for recent reviews). Adgrg6 (also known as Gpr126) is one of the better‐characterized aGPCRs. Initially described in mammals (Moriguchi et al., 2004; Stehlik, Kroismayr, Dorfleutner, Binder, & Lipp, 2004), it subsequently gained attention through the analysis of mutant phenotypes in the zebrafish (Figure 1) (Geng et al., 2013; Monk et al., 2009; Monk, Oshima, Jörs, Heller, & Talbot, 2011), originally identified through mutagenesis screens for morphology (Whitfield et al., 1996) or myelination (Pogoda et al., 2006).

Adgrg6 has a conserved role in the formation of the myelin sheath that insulates axons of the peripheral nervous system (PNS) in vertebrates (Monk et al., 2009, 2011). The PNS is myelinated by neural‐crest‐derived Schwann cells, and Adgrg6 is essential for Schwann cell maturation and the initiation of myelination in both zebrafish and mammals (Monk et al., 2009, 2011). Homozygous *adgrg6* ^*−/−*^ mutant zebrafish show a reduction or loss of the Schwann cell marker gene *myelin basic protein* (*mbp*) in the periphery (Figure 1b) (Geng et al., 2013; Monk et al., 2009). In the PNS, *adgrg6* is normally expressed in Schwann cells (Monk et al., 2009): contact with the axon is thought to trigger signaling through a canonical Gα_s_ cascade, leading to activation of adenylyl cyclase and the production of cAMP, in turn activating Protein Kinase A and the expression of downstream target genes, including *oct6* and *egr2* (Monk et al., 2009).

In addition to a reduction or loss of peripheral myelination, *adgrg6* mutant zebrafish also have a striking inner ear phenotype (Figure 1) (Geng et al., 2013; Monk et al., 2009; Whitfield et al., 1996). Epithelial projections in the otic vesicle, which normally initiate the formation of the semicircular canal ducts, overgrow and fail to fuse in the mutant, resulting in an easily identifiable swelling of the ear (Diamantopoulou et al., 2019; Geng et al., 2013). As in the PNS, Adgrg6 signaling in the developing zebrafish ear appears to be triggered by cell–cell contact and to act through cAMP, but here *adgrg6* is expressed on both sides of the fusing tissue (Geng et al., 2013). Many genes, including those coding for extracellular matrix (ECM) proteins and synthases, together with various developmental signaling pathway components, remain expressed at abnormally high levels in the *adgrg6* ^*−/−*^ mutant ear (Geng et al., 2013). These findings suggest that Adgrg6 signaling primarily acts (directly or indirectly) to repress gene expression in the developing ear.

The *adgrg6* gene is expressed dynamically in the developing embryo. In the zebrafish, the ear is a major site of expression, but *adgrg6* is also expressed in the neural crest and its derivatives (Schwann cells, craniofacial cartilage), the heart, mesoderm, and other tissues (Geng et al., 2013; Monk et al., 2009; reviewed in Patra, Monk, & Engel, 2014). In the 1‐day‐old zebrafish embryo, the expression pattern is very similar to that of the transcription factor gene *sox10*, and *adgrg6* expression in the ear and neural crest (but not in the heart) is reduced in *sox10* ^*−/−*^ mutants, suggesting that Sox10 may be an upstream regulator of *adgrg6* expression in these tissues (Geng et al., 2013). In mammals, *Adgrg6* is known to be expressed in multiple tissues, including in the adult (Mogha et al., 2016; Musa et al., 2019; Patra et al., 2014; Waller‐Evans et al., 2010).

The zebrafish *adgrg6* mutations identified or generated to date form an allelic series (Table 1) that has helped to dissect protein function. The different variants include hypomorphic missense mutations involving single amino acid changes in the transmembrane domain, which have been exploited in chemical screening experiments (see below), and point mutations that introduce stop codons early in the coding sequence, resulting in stronger ear and myelination defects (Figure 1). However, even strong *adgrg6* mutations in zebrafish are homozygous viable, unlike in the mouse, where targeted disruption of *Adgrg6* is lethal before or soon after birth (Monk et al., 2011; Waller‐Evans et al., 2010). Although this may reflect differences in Adgrg6 function between the two species, the effects of complete deletion of the zebrafish gene have not yet been demonstrated. It is also possible that the murine gene, which is expressed in the placenta, has a role in the development of extraembryonic tissues, which may contribute to the observed lethality in mutants (Waller‐Evans et al., 2010).

**TABLE 1:** List of key zebrafish *adgrg6* mutations

Mutation	Amino Acid	Domain	Reported phenotype	Reference
*stl47*	△5+3, N68K, fs*28	CUB	ear, radial sorting, PNS myelination	(Petersen *et al*., 2015)
*stl464*	*D134*A, *F135*A	CUB	ear & PNS myelination	(Leon et al., 2020)
*fr24*	L463*	SEA	ear & PNS myelination	(Geng et al., 2013)
*st49*	Y782*	GAIN	ear & PNS myelination	(Monk et al., 2009; Pogoda et al., 2006)
*vu39*	W804*	GPS	ear & PNS myelination	(Geng et al., 2013)
*stl215*	△*G831*, *I832*	*Stachel*	ear & PNS myelination	(Liebscher, Schön, et al., 2014)
*st63*	*C917*Y	TM2	PNS myelination	(Monk et al., 2009; Pogoda et al., 2006)
*tb233c*	*I963*N	TM4	ear & PNS myelination	(Geng et al., 2013; Whitfield et al., 1996)
*tk256a*	*P969*L	TM4	ear & PNS myelination	(Geng et al., 2013; Whitfield et al., 1996)

**FIGURE 2:**
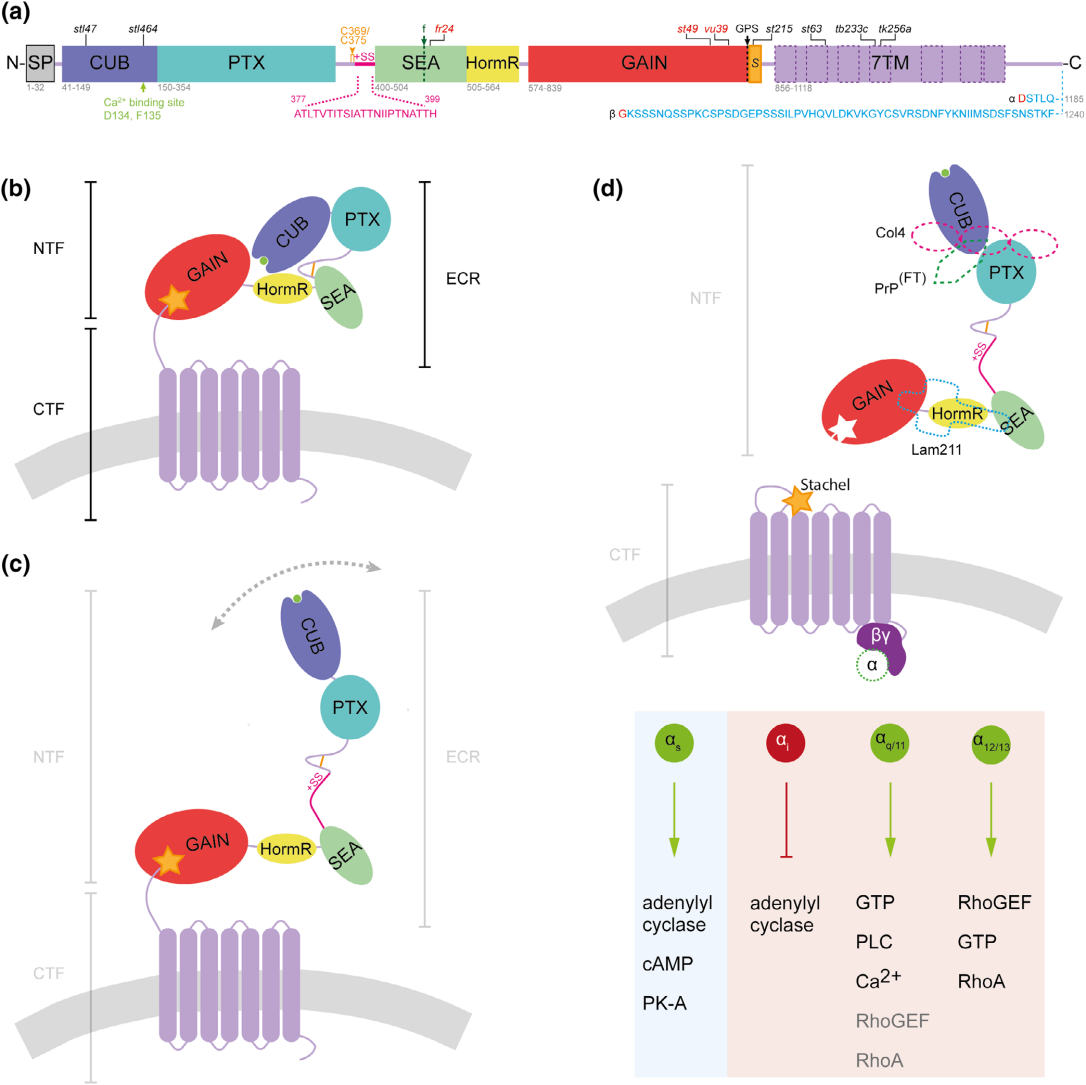
Schematic models of zebrafish Adgrg6 protein structure and mechanism of activation. (a) Zebrafish Adgrg6 protein sequence and domain organization, drawn to scale: signal peptide (SP), Complement C1r/C1s, Uegf, BMP1 (CUB), Pentraxin (PTX), Sperm protein, Enterokinase and Agrin (SEA), hormone receptor (HormR), GPCR autoproteolysis‐inducing (GAIN), *Stachel* sequence (S) and 7‐pass transmembrane (7TM, magenta) domains. The sequence of Adgrg6 splice isoforms is shown below the diagram: S1, including exon 6 (+SS, pink), and the short (α) and long (β) isoforms resulting from inclusion or exclusion, respectively, of exon 25 (amino acid sequence in blue; splice site in red). The two cysteine residues (C369, C375) that form a bond in the linker between PTX and SEA domain are shown in amber. Cleavage sites are highlighted by dotted lines: GPCR autoproteolytic site (GPS, black), and position of furin cleavage site in mammalian ADGRG6 (f, green). Positions of widely studied zebrafish mutations are shown above the diagram: truncating (nonsense) mutations in red; missense mutations in black. (b) Structure of Adgrg6 S2 isoform in closed conformation (not to scale). Domains correspond to those illustrated in (b); Ca^2+^ − binding site (green) within CUB domain, cell membrane (gray). (c) Structure of Adgrg6 S1 isoform in open conformation. (d) Overview of potential Adgrg6 *Stachel* activation mechanism (not to scale). Tethered *Stachel* sequence (amber star) self‐activates Adgrg6 following removal of NTF. The NTF is also thought to signal in trans (not shown). Known Adgrg6 NTF ligands (dashed shapes) include Collagen IV (pink, Col4), prion protein flexible tail (PrP^FT^, green), and Laminin‐211 (Lam211, turquoise). Signaling is transduced by various G protein α subunits activating intracellular pathways, elucidated from zebrafish Adgrg6 studies (light blue) and mouse or human ADGRG6 studies (light red). Diagrams are adapted from (Leon et al., 2020); the figure summarizes findings from this study and from (Geng et al., 2013; Küffer et al., 2016; Liebscher, Schön, et al., 2014; Lizano, Hayes, & Willard, 2021; Moriguchi et al., 2004; Paavola, Sidik, Zuchero, Eckart, & Talbot, 2014; Patra et al., 2013; Petersen et al., 2015)

Amino acid sequence and numbering are based on the reference sequence NM_001163291.2, with the exception of W804, which was reported as C804 in the reference sequence (see discussion in Geng et al. 2013). N68 was reported as Q68 in Petersen et al. 2015. Italic text indicates amino acid identity or similarity with the human protein. Abbreviations: △, deletion; fs, frame shift; see Figure 2 for domain name abbreviations.

Given the importance of *ADGRG6* in the myelination of the PNS, and its dynamic expression during development and in adult tissues, it is not surprising that *ADGRG6* also has a role in human disease. Evidence associating *ADGRG6* variants with a wide range of human pathologies, including both rare congenital conditions and more common disorders, is accumulating from family studies, GWAS data, RNA expression profiling studies, and animal models. In this review, we highlight some of the recent advances in Adgrg6 research, with a focus on the zebrafish model. We illustrate how the use of a suite of genetic, transgenic, imaging, and gene editing tools, together with small molecule screening approaches, make the zebrafish such a versatile model for the dissection of gene function, leading to new insights into the biology of the Adgrg6 receptor and the disorders that can result from its loss of function.

## 2 THE ADGRG6 GENE AND PROTEIN

### 2.1 *Adgrg6* gene structure and alternative splicing

Although aGPCRs are found throughout the metazoa, *Adgrg6* has no orthologue in the commonly used invertebrate model organisms *Drosophila* and *C. elegans*, the invertebrate chordates *Ciona* and amphioxus, or in jawless vertebrates such as lamprey and hagfish (Waller‐Evans et al., 2010; ensembl.org GRCz11; Yates et al., 2019). The *Adgrg6* gene thus appears to be specific to jawed vertebrates, and is evolutionarily conserved between teleost fish, amphibians, reptiles, birds, and mammals (ensembl.org GRCz11). Within the aGPCR class, Adgrg6 is most closely related to secretin‐like GPCRs (reviewed in Patra et al., 2014; Scholz, Langenhan, & Schöneberg, 2019). The zebrafish *adgrg6* gene has 26 exons and spans almost 90 kb. Unlike many zebrafish genes, which have two isoforms as a result of a teleost whole‐genome duplication event (Meyer & Schartl, 1999), zebrafish *adgrg6* has a 1:1 correspondence—and ~50% nucleotide identity—with the orthologous human gene *ADGRG6*. The zebrafish and human Adgrg6 proteins also show nearly 50% identity at the amino acid level, with the highest levels of conservation in the 7‐transmembrane domain and the CUB domain of the ECR (Leon et al., 2020; ensembl.org GRCz11; Yates et al., 2019).

Alternative splicing is a feature of aGPCR genes (Salzman et al., 2016). The expression of multiple isoforms, particularly of the ECR, is thought to influence mechanical interactions with binding partners, enhance receptor versatility and offer a regulatory mechanism for receptor activity (Bjarnadóttir et al., 2007). The human and zebrafish *ADGRG6* genes are known to have four main alternative splice forms (Moriguchi et al., 2004; Patra et al., 2014). These include inclusion (S1 form, also known as +ss) or exclusion (S2 form, −ss) of the short exon 6, which codes for 23 amino acids in the zebrafish Adgrg6 ECR (Patra et al., 2014). In addition, the presence or absence of the penultimate exon results in a frameshift that alters the coding sequence for the intracellular C‐terminal domain, which could potentially influence downstream signaling events. Adhesion GPCRs in general are found to have a high level of somatic mutations (Kan et al., 2010; O'Hayre et al., 2013) and the human *ADGRG6* gene in particular has numerous variants (35,447 variants; ADGRG6‐201 transcript, Yates et al., 2019).

### 2.2 Adgrg6 protein structure

Current models for the zebrafish Adgrg6 protein structure are shown in Figure 2. During biosynthesis, autoproteolytic cleavage at the GPCR Proteolysis Site (GPS) in the GAIN domain separates the protein into N‐ and C‐terminal fragments (NTF, CTF), which can remain non‐covalently associated at the membrane (Araç et al., 2012). The NTF is composed of structural domains including the Complement C1r/C1s, Uegf and Bmp1 (CUB), Pentraxin (PTX), and Hormone Receptor (HormR) domains (Araç et al., 2012; Moriguchi et al., 2004), together with a recently identified sperm protein, enterokinase and agrin (SEA) domain (Leon et al., 2020). The SEA domain contains a furin cleavage site in the human and mouse protein that is not conserved in zebrafish. Like other aGPCRs, Adgrg6 undergoes post‐translational modifications including N‐linked glycosylation of the ECR, which is thought to contribute to the adhesive properties of the NTF (Langenhan, Aust, & Hamann, 2013; Leon et al., 2020; Patra et al., 2014).

Determining the structure of the receptor, and in particular that of the ECR, is fundamental to understanding its mechanism of action. The crystal structure of the zebrafish Adgrg6 ECR is one of only four aGPCR protein structures determined to date (Leon et al., 2020; Ping et al., 2021; Salzman et al., 2016; Vakonakis, Langenhan, Prömel, Russ, & Campbell, 2008), and has highlighted how conformational changes of the ECR might regulate receptor activity. Crystallization of the zebrafish Adgrg6 ECR and analysis with a range of imaging techniques has revealed that the S2 form adopts a closed conformation, in which the CUB domain interacts with the HormR domain (Figure 2b) (Leon et al., 2020). This closed configuration is dependent on the presence of a proposed Ca^2+^ binding site within the CUB domain, together with a disulfide‐stabilized loop between the SEA and PTX domains. The loop inserts between the CUB and HormR domains, stabilizing their interaction (Leon et al., 2020). The 23‐amino‐acid linker in the S1 form appears to disrupt the stability of the CUB‐HormR interaction, and the S1 ECR shows a variety of conformations, indicating an ability to switch between an open and closed state (Figure 2c) (Leon et al., 2020). This mobility translates to an increase in Adgrg6 signaling in comparison with the basal activity associated with the closed isoform in an in vitro cAMP assay (Leon et al., 2020).

An intact Ca^2+^‐binding site is also necessary for optimal receptor function. A conserved aspartate residue co‐ordinates a calcium ion in Ca^2+^‐binding CUB domains from different aGPCRs (Gregory, Thielens, Arlaud, Fontecilla‐Camps, & Gaboriaud, 2003). Disruption of the site in the zebrafish Adgrg6 protein, by CRISPR/Cas9‐mediated replacement of both D134 and F135 residues with alanines, induces ear and myelination defects similar to those observed in a strong loss‐of‐function *adgrg6* mutant (Leon et al., 2020). In the crystallized ECR, disruption of the Ca^2+^‐binding site resulted in an open configuration, but despite this, the S1 Adgrg6 isoform was unable to mediate an increase in cAMP accumulation above basal levels (Leon et al., 2020). These findings propose a complex mechanism by which the ECR may regulate Adgrg6 function, with an intact Ca^2+^‐binding site and the 23‐amino acid linker being necessary to trigger a boost in Adgrg6 signaling.

### 2.3 Adgrg6 ligands and mechanosensing

The multiple domains in the long ECRs of aGPCRs provide the opportunity to bind to many different partners. Studies in a range of model systems and contexts, including morpholino phenocopy and mutant rescue experiments in the zebrafish, indicate that the Adgrg6 NTF interacts with the ECM components Collagen IV (Paavola et al., 2014) and Laminin‐211 (Petersen et al., 2015), and the flexible tail residues 23–50 of the Prion protein (PrP_23‐50_) (Küffer et al., 2016) (Figure 2d). However, it is not fully understood how Adgrg6 and these natural ligands operate at the cell membrane in vivo. Collagen IV and the Prion protein appear to mediate cAMP accumulation through binding to the Adgrg6‐NTF directly (Küffer et al., 2016; Paavola et al., 2014). However, application of purified Laminin‐211 to cells transfected with human ADGRG6 resulted in an unexpected decrease in cAMP accumulation, attributed to an inhibition of basal signaling activity (Petersen et al., 2015). In this experimental context, receptor activation required the application of force, by sample vibration, to mimic the in vivo environment at the cell membrane (Petersen et al., 2015). These observations suggest a variety of mechanisms through which NTF binding partners can activate Adgrg6 signaling, and a potential role for Adgrg6 in mechanosensing. Indeed, a study analyzing expression domains of a LacZ reporter for *Adgrg6* expression in the mouse has highlighted its predominant expression in mechanosensing tissues (Musa, Cazorla‐Vázquez, et al., 2019).

Adapting natural ligands as agonists of the Adgrg6 signaling pathway could have exciting therapeutic potential for human diseases involving disruption of ADGRG6 function. However, recent work to develop the Prion protein (PrP) as an Adgrg6 agonist has proved challenging. The application of a stable Adgrg6‐activating PrP‐based molecule failed to rescue a peripheral demyelinating neuropathy in PrP knock‐out mice, and transcriptomic data suggested that the rescuing agent might itself be myotoxic (Henzi et al., 2020). Further identification of NTF ligands will be critical in teasing out the underlying mechanisms leading to Adgrg6 activation. However, natural ligands may not present a viable approach for therapeutic design due to their widespread effects in vivo.

### 2.4 Tethered agonist signaling: Autoactivation of the Adgrg6 receptor

Early aGPCR studies highlighted that the NTF‐CTF interaction at the cell membrane provides an inhibitory control mechanism to limit receptor signaling through G proteins (Okajima, Kudo, & Yokota, 2010; Paavola, Stephenson, Ritter, Alter, & Hall, 2011; Ward et al., 2011). Utilizing cAMP assays as a measure of ADGRG6 activity, Liebscher et al. (2014) uncovered a tethered peptide agonist, terming it the “*Stachel”* (“stinger”) sequence. The peptide is located at the N‐terminal end of the CTF, where it is embedded in beta sheets of the GAIN domain (Figure 2) (Araç et al., 2012; Beliu et al., 2021). Although direct binding has not yet been visualized, in vivo treatment of hypomorphic *adgrg6* ^*st63*^ zebrafish larvae with a synthetic 16‐amino‐acid *Stachel* peptide has been shown to mediate some restoration of Schwann cell *mbp* expression in the PNS (Liebscher, Schön, et al., 2014). A *Stachel* sequence has since been identified in multiple other aGPCRs (Demberg, Rothemund, Schöneberg, & Liebscher, 2015; Stoveken, Hajduczok, Xu, & Tall, 2015), suggesting it may be a common mechanism of action.

Two different models have been proposed for *Stachel* activation of signaling. The first relies on the physical removal of the NTF—through ligand interactions—to expose the tethered agonist, enabling it to bind to the active site of the CTF and self‐activate receptor signaling (Liebscher, Schön, et al., 2014; Petersen et al., 2015). However, a recent study using molecular dynamic simulations of aGPCRs has proposed an alternative mechanism, in which the *Stachel* sequence is transiently exposed through structural flaps within the GAIN domain, following moderate movements (Beliu et al., 2021). Although not confirmed for Adgrg6, this notion could explain the basal activity of Adgrg6 that is associated with the S2 isoform (Leon et al., 2020).

*Stachel*‐independent activation of aGPCRs has also been detected by multiple groups (Kishore, Purcell, Nassiri‐Toosi, & Hall, 2016; Salzman et al., 2017). Cells expressing a mutant isoform of ADGRG1 (H381S), defective in GPS autoproteolysis, exhibit an increase in signaling following exposure to monobody ligands, a response that is unaffected by disruption of the *Stachel* sequence (Salzman et al., 2017). Furthermore, cells expressing an engineered form of ADGRB1 lacking the *Stachel* peptide exhibit differential signaling behavior, including elevated TGFα shedding, in comparison with the intact receptor (Kishore et al., 2016). Exploiting equivalent Adgrg6 mutant isoforms may reveal further evidence of this mechanism among aGPCRs and in which tissues or developmental contexts different mechanisms apply.

### 2.5 Adgrg6 coupling to G proteins

Initial studies to identify the G protein or proteins that couple to zebrafish Adgrg6 showed that the myelination defects in *adgrg6* ^*st49*^ mutants could be rescued by treatment with forskolin, an adenylyl cyclase agonist that raises intracellular cAMP levels (Monk et al., 2009). The ear phenotype in *adgrg6* ^*tb233c*^ mutants can also be ameliorated by treatment with either forskolin or 3‐isobutyl‐1‐methylxanthine (IBMX), a phosphodiesterase inhibitor that prevents cAMP degradation (Diamantopoulou et al., 2019; Geng et al., 2013). These findings suggest that Adgrg6 couples to a Gα_s_ signaling pathway in both Schwann cells and in the ear (Figure 2d). Coupling to Gα_s_ has since been confirmed by several groups by detecting changes in intracellular cAMP levels following expression of human ADGRG6 (Liebscher, Schön, et al., 2014; Paavola et al., 2014; Petersen et al., 2015). Nevertheless, this may not represent the full picture as aGPCRs are well‐known to signal through multiple G‐protein pathways (reviewed in Langenhan, 2019). Application of a novel enterokinase‐activated tethered ligand system of ADGRG6 not only confirmed coupling to Gα_s_, but also demonstrated stimulation of reporters for serum response factor (SRF) and serum response element (SRE), indicating coupling to Gα_12/13_ and Gα_q/11_ pathways, respectively (Lizano et al., 2021). However, a study utilizing chimeric G proteins concluded that the human receptor can couple to Gα_s_ and Gα_i_, but not Gα_q_ (Mogha et al., 2013). Given the evidence, a complex interplay of multiple signaling pathways is likely to be associated with the Adgrg6 receptor in vivo.

### 2.6 Signaling via the Adgrg6 N‐terminal fragment

The NTF produced by autoproteolytic cleavage of Adgrg6 at the GPS, or potentially through furin cleavage of the human or mouse protein, has been shown to act in trans, independently of the CTF. Initial studies of *Adgrg6* knock‐out mice uncovered a delay in radial sorting of peripheral axons by Schwann cells, suggesting a role for Adgrg6 in this process (Monk et al., 2011). Axonal sorting deficiencies were confirmed in zebrafish by analyzing *adgrg6* ^*stl47*^ mutants, which predict a protein with a truncated CUB domain (Petersen et al., 2015). Moreover, the *stl47* mutants were unresponsive to forskolin treatment, suggesting that axonal sorting materializes independently of the canonical G‐protein‐coupled signaling associated with the Adgrg6 CTF. Laminin‐211 was identified as a ligand of the Adgrg6 NTF in axonal sorting, and multiple zebrafish *adgrg6* mutant alleles were used to characterize this interaction (Petersen et al., 2015). Given that Collagen IV can also bind the NTF (Paavola et al., 2014), and that *Col4a1* mouse mutants display peripheral radial sorting defects (Labelle‐Dumais et al., 2019), it is likely that Collagen IV performs a similar function to Laminin‐211 in this context in the mouse.

Further evidence for the NTF acting in a cAMP‐independent manner comes from studies in mice, where conditional disruption of *Adgrg6* in chondrocyte lineages results in skeletal abnormalities that mimic human disorders, as discussed in Section 3 below. Treatment of *Adgrg6* conditional knock‐out mice with the phosphodiesterase inhibitor Rolipram, which would be expected to restore cAMP signaling downstream of the receptor, was unable to rescue the skeletal phenotype, suggesting alternative signaling pathways may be required in mammalian chondrocytes (Karner, Long, Solnica‐Krezel, Monk, & Gray, 2015). In another study, over‐expression of the Adgrg6 NTF (S1 isoform) was reported to delay ossification of human mesenchymal stem cells in vitro; the authors propose that differential expression of *ADGRG6* or its isoforms could contribute to the abnormal curvature of the spine in patients with Adolescent Idiopathic Scoliosis (Xu et al., 2019) (see also Section 3.4).

The Adgrg6 NTF has also been implicated in heart development. *Adgrg6* is expressed in the mouse and zebrafish heart (Geng et al., 2013; Monk et al., 2011; Moriguchi et al., 2004; Patra et al., 2013), and homozygous mutant mice have cardiac hypotrabeculation defects (Patra et al., 2013). However, cardiovascular defects are not obvious in most of the zebrafish mutant alleles analyzed to date (Geng et al., 2013; Leon et al., 2020; Monk et al., 2009), and *adgrg6* homozygous mutant fish are adult viable. By contrast, morpholino‐mediated knock‐down of *adgrg6* was reported to result in a similar hypotrabeculation defect to that in the mouse mutants, which could be rescued by injection of mRNA coding for the NTF (Patra et al., 2013). The reported heart defects in zebrafish morphants could be the result of an off‐target effect of the morpholino, but it is also possible that genetic compensation—now well characterized for other zebrafish genes (El‐Brolosy et al., 2019)—acts to mask any heart phenotype in the mutants. Nevertheless, the mutant alleles with no overt heart defects still predict that a truncated NTF could be produced. The most severe truncating allele, *stl47*, predicts a deletion of most of the NTF; these mutants do display heart oedema (Petersen et al., 2015), and a recent report suggests they also have hypotrabeculation (Srivastava et al., reviewed in Morgan et al., 2019). A separate report found *Adgrg6* expression downstream of Notch signaling in both trabeculation and compaction of the mouse heart, with a reduction in cardiac *adgrg6* expression in the zebrafish Notch pathway mutant *mind bomb (E3 ubiquitin ligase)* (D'Amato et al., 2016). In addition, injection of zebrafish embryos with a potential post‐transcriptional regulator of Adgrg6, *miR‐27b*, produced the same hypotrabeculation phenotype as in *adgrg6* morphants (Musa, Srivastava, Petzold, Cazorla‐Vázquez, & Engel, 2019). Given these observations, a detailed analysis of the cardiac phenotype in the different zebrafish alleles will be important to confirm any role for Adgrg6 in zebrafish heart trabeculation.

## 3 ADGRG6 AND HUMAN DISEASE

### 3.1 *ADGRG6* coding sequence mutations and inherited disease

Many of the mutations in *ADGRG6* associated with human disease (Table 2) affect residues that are conserved in zebrafish. The first studies to implicate ADGRG6 in human disease corroborated the myelination defects discovered in zebrafish and mouse mutants. Probands from three consanguineous families with Lethal Congenital Contracture Syndrome 9 (LCCS9), a rare form of Severe Arthrogryposis Multiplex Congenita (AMC), were found to harbor different homozygous recessive mutations in the *ADGRG6* coding sequence (Ravenscroft et al., 2015). AMC is known to have multiple causes that can be musculoskeletal or neuronal in origin. Contractures were observed in the limbs of mouse *Adgrg6* mutants that lack myelination of the peripheral nerves (Monk et al., 2011). In the Ravenscroft et al. (2015) study, a lack of MBP staining was also observed in the intramuscular nerves, confirming that a myelination defect underlies the phenotype. In a separate study of an Iranian family with autosomal recessive Intellectual Disability, whole exome sequencing identified a strong candidate missense mutation in the transmembrane domain of *ADGRG6*. The two patients survived beyond puberty and displayed a number of common features with LCCS9, including contractures (Hosseini et al., 2018).

**TABLE 2:** List of key *ADGRG6* coding variants associated with human disease conditions

Disease/condition	Amino acid	Domain	Mutation (SNP reference)	Reference
LCCS9	R7*	SP	Nonsense homozygous	(Ravenscroft et al., 2015)
LCCS9	Q716T, fs*16	GAIN	Duplication c2144dup homozygous	(Ravenscroft et al., 2015)
LCCS9	*V769*E	GAIN	Missense homozygous	(Ravenscroft et al., 2015)
Intellectual disability	*W1088*C	TM6–7 extracellular loop	Missense homozygous	(Hosseini et al., 2018)
Periodontitis	*R1057*Q	TM5–6 intracellular loop	Missense (rs536714306)	(Kitagaki et al., 2016)
COPD	*S123*G, K230Q	CUB, PTX	Missense (rs17280293)	(Terzikhan et al., 2018)
Hypobaric hypoxia adaptation	*S123*G	CUB	Missense (rs17280293)	(Eichstaedt et al., 2017)
Pulmonary function	K230Q	PTX	Missense (rs11155242)	(Hancock et al., 2010)

*Note:* Amino acid sequence and numbering are based on the reference sequence NM_198569.3. Italic text indicates amino acid identity or similarity with the zebrafish protein.

Abbreviations: COPD, chronic obstructive pulmonary disease; fs, frame shift. See Figure 2 for domain name abbreviations.

### 3.2 Identification of *ADGRG6* variants through GWAS and RNA sequencing

Recent Genome‐Wide Association Studies (GWAS) and RNAseq studies have found many associations with variants in both coding and non‐coding regions of the human *ADGRG6* locus that may provide clues to ADGRG6 function in disease (see https://www.ebi.ac.uk/gwas/genes/ADGRG6 for a comprehensive list). One example is a missense mutation in the *ADGRG6* transmembrane region, R1057Q, that has been linked to severe periodontitis in a cohort of Japanese patients (Kitagaki et al., 2016). In vitro studies found that the amino acid change reduced cAMP accumulation compared with wild‐type ADGRG6; expression of downstream target genes (*Bmp2*, *ID2* and *ID4*) and cytodifferentiation were affected. This work implicates ADGRG6 in the homeostasis of periodontal ligament tissues, making the R1057Q mutation a strong candidate as a functional cause of the disease.

The mammalian *ADGRG6* gene is widely expressed in the lung (Musa, Cazorla‐Vázquez, et al., 2019) and evidence is increasing for the association of *ADGRG6* variants in both coding and intronic regions with different aspects of lung function, including chronic obstructive pulmonary disease (COPD) (Hancock et al., 2010; Jackson et al., 2016; Soler Artigas et al., 2015; Wilk et al., 2012). One of these variants, resulting in the missense mutation S123G, was also identified in Andean people living at altitude, suggesting it may be involved in the physiological adaptation to hypobaric hypoxia (Eichstaedt et al., 2017). A separate analysis of this variant found that expression of *ADGRG6* in human lung tissue was decreased in COPD patients and in individuals with decreased pulmonary ventilatory function, suggesting this variant may also be functional (Terzikhan et al., 2018).

*ADGRG6* variants and transcriptional changes have also been associated with various human cancers. A transcriptomic analysis of 772 GPCRs in 148 acute myeloid leukemia (AML) samples, encompassing different subgroups, identified *ADGRG6* as one of 19 down‐regulated GPCRs, with others including the closely related *ADGRG1* and *SMO* (Maiga et al., 2016). However, *ADGRG6* was up‐regulated in specific MLL translocations, suggesting ADGRG6 as a candidate disease‐specific therapeutic target in these sub‐groups (Maiga et al., 2016). The *ADGRG6* locus also contains a highly mutable palindromic motif in intron 6 that is found in 2.7% of breast cancers (Nik‐Zainal et al., 2016) and in more than 45% of bladder cancers (Garinet et al., 2019). *ADGRG6* is expressed in the bladder and mutations in the intron 6 motif correlated with an increase in *ADGRG6* expression and a poor prognosis in one study (Wu et al., 2019). Increased *ADGRG6* expression also correlated with an increase in tumor angiogenesis (Wu et al., 2019), supporting previous evidence that ADGRG6 may have a role more generally in angiogenesis via the VEGF signaling pathway (Cui et al., 2014; Stehlik et al., 2004).

### 3.3 Role of ADGRG6 in the skeleton

Noncoding variants at the human *ADGRG6* locus are associated with different aspects of musculoskeletal development, including height (Soranzo et al., 2009; J. Zhao et al., 2010) and adolescent idiopathic scoliosis (AIS) (Karner et al., 2015; Kou et al., 2013, 2018; Qin et al., 2017; J. F. Xu et al., 2015). AIS is very common, affecting up to 3% of children in the UK (Lenssinck et al., 2005). In mice, *Adgrg6* is expressed in chondrogenic lineages of the axial skeleton (Liu et al., 2019). *Adgrg6* knock‐out mice are shorter than their WT litter‐mates and have spinal abnormalities (Monk et al., 2011). Although this suggests a functional role in skeletal development, scoliosis can occur secondary to neuropathy or can be co‐morbid with joint contractures, as is found in AMC (Ravenscroft et al., 2015).

To address whether skeletal defects are the underlying cause of the AIS phenotype in *Adgrg6* mutants, two studies used tissue‐specific deletion of *Adgrg6* in mice (Karner et al., 2015; Sun et al., 2020). Conditional knock‐out of *Adgrg6* in osteoblasts did not result in scoliosis; instead, mutant mice showed delayed ossification and reduced growth (Sun et al., 2020), supporting a role for *ADGRG6* in influencing body length. This study identified Collagen IV as the Adgrg6 ligand and found that treatment with forskolin could partially rescue the phenotype (Sun et al., 2020). In contrast to the deletion of *Adgrg6* in osteoblasts, deletion of *Adgrg6* in chondrocyte lineages of the spine in mice resulted in abnormalities of the intervertebral disc (IVD), including mechanical stiffening and eventual disc herniation, or scoliosis, depending on the transgenic driver used (Karner et al., 2015; Liu et al., 2019). Expression of *Stat3*, a gene known to be upregulated in disc degeneration and osteoarthritis, was increased (Liu et al., 2019). A STAT3 inhibitor used to treat osteoarthritis protected against the defect, suggesting that ADGRG6 is a potential therapeutic target for IVD degeneration. Other studies also implicate Adgrg6 in AIS pathology (Xu et al., 2019; Xu, Lin, et al., 2019), but analysis of the *ADGRG6* transcript expression profile in both normal and AIS patient tissue is needed to confirm these results. In the zebrafish, *adgrg6* is expressed in the developing craniofacial and axial skeleton (Geng et al., 2013; Glenn & Talbot, 2013). Delayed ossification and a slight reduction in body length has also been reported in zebrafish *adgrg6* morphants (Kou et al., 2013); however, a difference in body length has not been reported in any of the zebrafish *adgrg6* mutant alleles. Further work is needed to determine if *Adgrg6* plays a role in body length and IVD development in fish.

### 3.4 Role of Adgrg6 in remyelination and regeneration

Unlike mammals, zebrafish are able to regenerate a wide range of adult tissues following injury, including the spinal cord, fin and heart (reviewed in Cigliola, Becker, & Poss, 2020; Roehl, 2018; Sanz‐Morejón & Mercader, 2020). To date, most studies of Adgrg6 have focused on a role for the receptor in the initial development of Schwann cell myelination, rather than the maintenance or repair of myelination at adult stages (Glenn & Talbot, 2013). However, expression of *Adgrg6* is maintained and required in adult mammalian and zebrafish Schwann cells (Mogha et al., 2013; Monk et al., 2009), raising the possibility of a role for the receptor in peripheral nerve remyelination and regeneration following injury. Mogha et al. (2016) generated a Schwann‐cell‐specific, tamoxifen‐inducible *Adgrg6* knock‐out mouse model and performed a crush injury of the sciatic nerve. Twenty‐one days after nerve crush, the tamoxifen‐injected mice showed significantly impaired sciatic nerve remyelination and persistent myelin debris, in comparison with control‐injected animals (Mogha et al., 2016). These findings suggested that Adgrg6 is needed autonomously in mammalian Schwann cells for remyelination, but is also required non‐autonomously for recruitment of macrophages to clear damaged tissue (Mogha et al., 2016). Furthermore, length measurements of the longest regenerating axons (from the crush site) revealed that axon regeneration is greatly impaired in mutant mice compared with controls, proposing another non‐autonomous role for Adgrg6 in peripheral axon regeneration following nerve injury (Mogha et al., 2016). Adgrg6 is both expressed and required in non‐myelinating terminal Schwann cells for re‐innervation following nerve crush at the neuromuscular junction, where it also has a non‐cell‐autonomous role in the immune response after injury (Jablonka‐Shariff, Lu, Campbell, Monk, & Snyder‐Warwick, 2020). In the zebrafish, a study using the *stl47* allele suggests that Adgrg6 is not required for the initial changes in Schwann cell morphology in response to peripheral motor nerve transection (Ducommun Priest, Navarro, Bremer, & Granato, 2019). However, a full analysis of nerve regeneration in zebrafish *adgrg6* mutants has not yet been performed, to our knowledge.

## 4 ADGRG6 AND DRUG DISCOVERY

### 4.1 GPCRs as druggable targets

Compounds targeting GPCRs constitute a major class in the global market share of therapeutic drugs, reflecting the diverse roles of these receptors in cellular physiology, accessibility at the cell surface, and the presence of binding pockets within their structure (Manglik & Kruse, 2017). Adhesion GPCRs, however, are not represented as targets for licensed drugs, but offer similar potential, given their role in human disease. There are many challenges for aGPCR drug discovery, in part due to the multimodal nature of aGPCRs, which have functions in different tissues, and the possibility of polypharmacology, due to structural similarities between aGPCRs, with any drug identified potentially acting on multiple aGPCRs. In particular, the gedunin class of partial agonists can also act on multiple receptors (Stoveken, Larsen, Smrcka, & Tall, 2018).

Knowledge of the structure of the Adgrg6 ECR (Leon et al., 2020) should facilitate drug design; however, the structure of the CTF, and in particular of the pocket where the *Stachel* peptide binds, are still uncharacterised. The Adgrg6 ECR has structural similarities with the extendable ECR of the epidermal growth factor receptor (EGFR) (Leon et al., 2020). Cetuximab, an anti‐cancer monoclonal antibody drug, targets the ECR of EGFR, preventing its extension to the active isoform (Li et al., 2005). Leon et al. (2020) speculate that the dynamic Adgrg6 S1 ECR may be druggable through an equivalent approach to modulate mechanosensory and/or signal transducing functions. Although there are challenges for targeting ADGRG6, small molecule modulators have potential for therapeutic use against many ADGRG6‐disease linked conditions, including cancers where *ADGRG6* overexpression may contribute to the disease (see Section 3).

### 4.2 Small modulators of GPCRs as biological tools

In addition to therapeutics, chemical ligands that bind directly with the Adgrg6 protein could provide valuable tools to stabilize the receptor in an active or inactive conformation, which could facilitate isolation of the receptor in its in vivo conformation, or for the manipulation of pathway activity. Agonist and antagonist compounds are widely utilized as pharmacological tools to modulate the activity of key developmental signaling pathways, including those of the Hedgehog (Hh) and Wnt proteins, which signal via Smoothened and Frizzled family GPCRs, respectively (reviewed in Agostino & Pohl, 2020). A precedent for the translation of small molecules originally identified through developmental biological research to the clinic is the Smoothened inhibitor vismodegib, now a treatment for basal cell carcinoma (Ingham, 2018).

### 4.3 Zebrafish screens for small molecule modulators of Adgrg6 pathway activity

Zebrafish present an excellent whole‐animal model for precision medicine and are amenable to medium‐ and high‐throughput small molecule screening approaches (reviewed in Baxendale, van Eeden, & Wilkinson, 2017; Lam & Peterson, 2019). The multimodal nature of aGPCRs and the potential for polypharmacology present problems for traditional in vitro drug discovery pipelines. Adhesion GPCR function is very likely to be context‐dependent, due to, for example, interactions with the extracellular matrix and mechanosensing functions. In vivo screening naturally provides these physiological contexts, which are less likely to be recapitulated in an in vitro cell line‐based screening platform. The use of zebrafish drug screening approaches also has the advantage of eliminating compounds with toxic or off‐target effects in the primary screen.

**FIGURE 3:**
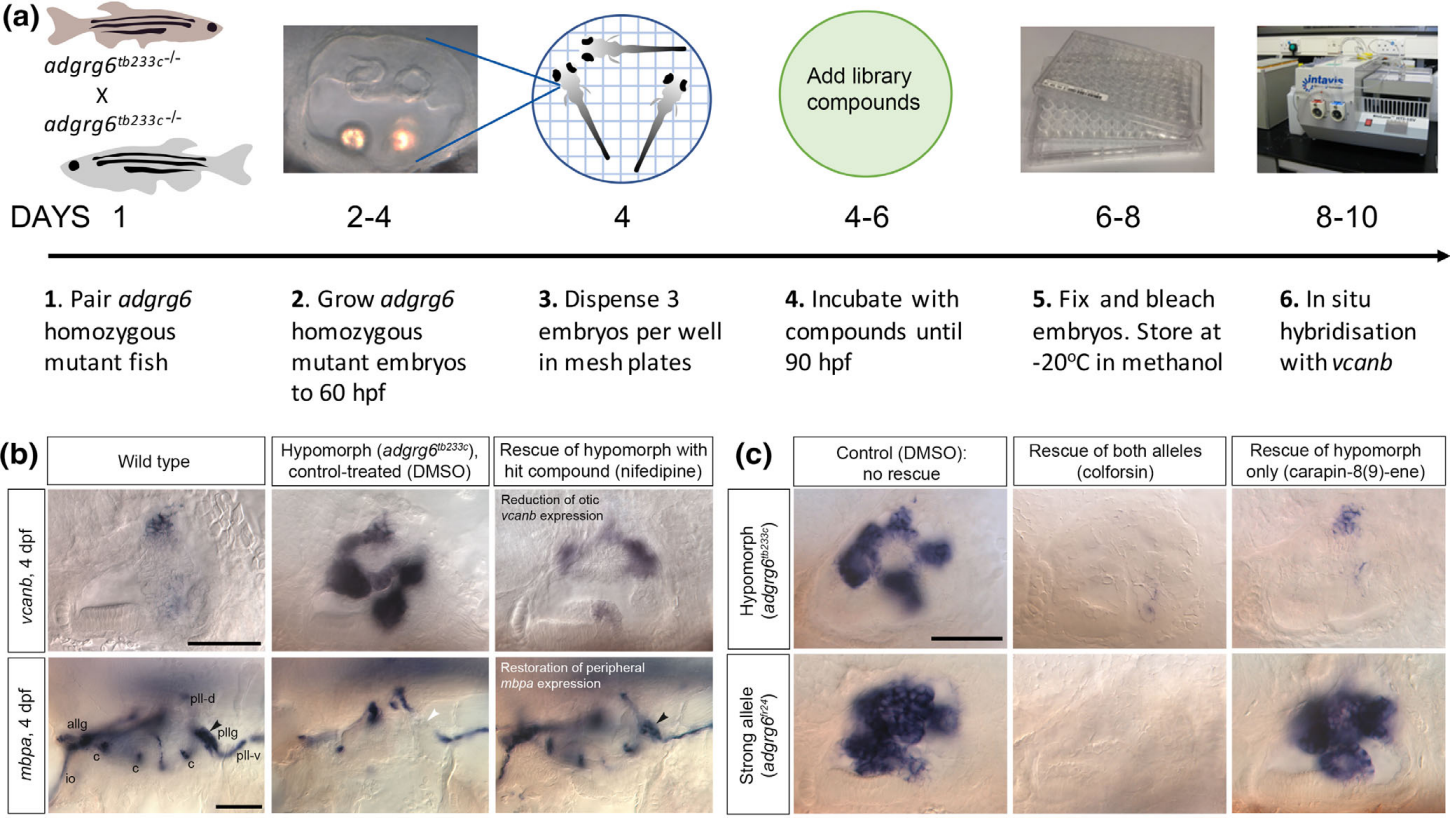
Design and proof‐of‐principle for a whole‐animal compound screen to identify agonists of the zebrafish Adgrg6 pathway. (a) Overview of screen pipeline. Batches of 100% homozygous embryos are dispensed into 96‐well plates, with three embryos (biological replicates) per well. Embryos are incubated in control and test compounds from a library of choice at an optimum time for rescue of the mutant phenotype. Effects of compounds on gene expression are measured by semi‐automated in situ hybridisation. (b) Example results from a two‐part in situ hybridization screen using the hypomorphic (*adgrg6* ^*tb233c−/−*^) alelle. Here, an example hit compound (nifedipine, a dihydropyridine) reduces *vcanb* expression in the ear (top panels) and restores *mpba* expression in Schwann cells of the lateral line nerves in the vicinity of the ear. Several areas of *mbpa* expression are rescued, in particular that associated with the posterior lateral line ganglion (pllg, arrowhead). Lateral views; anterior to the left, dorsal to the top. Abbreviations: allg, anterior lateral line ganglion; c, expression associated with the sensory cristae of the ear; DMSO, dimethyl sulfoxide; dpf, days post fertilization; io, infraorbital ramus of the anterior lateral line nerve; pll‐d, pll‐v, dorsal and ventral rami of the posterior lateral line nerve; pllg, posterior lateral line ganglion. Scale bars, 50 μm (top row); 50 μm (bottom row). (c) Use of hypomorphic and strong alleles to differentiate hit compound classes in the *vcanb* expression assay. Effects of example compounds are shown. Colforsin, a forskolin derivative, rescues both alleles efficiently, and is likely to act downstream of the pathway to raise cAMP levels. Carapin‐8(9)‐ene rescues the hypomorph, but has no effect on the stronger *fr24* allele. Compounds such as this may interact directly with the Adgrg6 receptor. Scale bar, 50 μm. Images reproduced from Diamantopoulou et al., 2019

Several features have made the zebrafish Adgrg6 pathway particularly advantageous for interrogation with small‐molecule‐based screens. Firstly, hypomorphic alleles—those with weak phenotypes—are amenable to modulation in two directions: rescue of the phenotype or its further exacerbation (Figure 3). Hypomorphs may also exhibit higher sensitivity to small‐molecule‐centered therapeutic approaches, as a weaker phenotype can sometimes be rescued more easily. Secondly, the available zebrafish *adgrg6* mutants are homozygous viable, allowing batches of 100% mutant embryos to be produced for screening assays. Thirdly, clear mRNA or transgene expression changes in the mutant have enabled the development of robust and reliable screening assays. Finally, the comparison of both hypomorphic and strong alleles in secondary screens has allowed the differentiation of different classes of hit compounds.

Two different screens have used these advantages in their approach (Bradley et al., 2019; Diamantopoulou et al., 2019). Using the expression of *versican* mRNA in the mutant ear as a phenotypic readout of Adgrg6 pathway activity, Diamantopoulou et al. (2019) identified several classes of small molecules that could rescue the otic phenotype in a*dgrg6* ^*tb233c*^ hypomorphic mutants, some of which also rescued the myelination defect (Figure 3). The hit compounds included colforsin (a forskolin derivative that raises cAMP levels), a group of dihydropyridines, and a cluster of gedunin derivatives (Diamantopoulou et al., 2019), some of which were independently identified as modulators of human ADGRG1 (Stoveken et al., 2018). Whereas colforsin was able to rescue both hypomorphic (*tb233c*) and strong (*fr24*) alleles, validating association of Adgrg6 to Gαs, other hit compounds were ineffective at rescuing the *fr24* allele (Diamantopoulou et al., 2019) (Figure 3). This latter class are of particular interest, as they may act directly at the level of the receptor. Further work will be needed to test these compounds in cell‐based cAMP or Ca^2+^ immobilization assays to determine direct agonism for Adgrg6 (Liebscher, Ackley, et al., 2014; Lizano et al., 2021).

Bradley et al. (2019) monitored changes in *mbp*‐driven transgene expression in Schwann cells for their compound screen in *adgrg6* ^*st63*^ hypomorphic mutants. They identified apomorphine hydrochloride, a dopamine receptor agonist, for its ability to induce upregulation of *mbp*‐driven GFP in *adgrg6* mutants and to mediate an increase in cAMP levels in Adgrg6‐expressing cells (Bradley et al., 2019). Differences in the hit molecules identified between this and the Diamantopoulou et al. (2019) screen are likely to reflect the different commercially‐available compound collections that were used, together with differences in the alleles used for screening and details of the screening assay design, including compound concentration and exposure time. Clearly, each study has yielded a rich resource of material for validation and further study. A novel approach that could provide further support for the action of apomorphine hydrochloride or other candidate compounds as Adgrg6 agonists is to determine coupling of mini‐G proteins to Adgrg6 following treatment (Carpenter & Tate, 2016; Nehmé et al., 2017). Such experiments would provide a direct measure of Adgrg6 activity following small molecule treatments, as opposed to the detection of downstream effector molecules, such as cAMP, that can be modulated by potential compound agonism on adenylyl cyclase.

Taken together, the research findings illustrate a space for multifaceted drug screening approaches that combine zebrafish phenotypic strategies with in vitro cell‐based assays to overcome the individual limitations of each in identifying aGPCR modulators. The availability of tailored technologies such as automated imaging for the zebrafish larva is improving the throughput of zebrafish small molecule screens (Bradley et al., 2019; Early et al., 2018). However, the lower‐cost semi‐quantitative in situ hybridization approach also remains effective (Diamantopoulou et al., 2019).

## 5 CONCLUSIONS AND FUTURE PERSPECTIVES

Recent research on Adgrg6 has led to a better understanding of this complex receptor, including its structure, mechanism of action, and roles in development and disease. Work in the zebrafish model has made significant contributions to this research effort. Nevertheless, many questions remain. For instance, we still need to know more about upstream regulators, downstream targets, ligand‐receptor binding and the different modes of signaling mediated by the tethered agonist and the NTF. However, there are many areas for future Adgrg6 research that play to zebrafish strengths in imaging, chemical screening, and gene editing.

One area where recent advances in technology can contribute is to understand the role that different *adgrg6* splice forms play in different developing tissues and how they lead to Adgrg6 protein diversity. At least four splice variants have been reported for the human gene, and it is likely RNAseq data will identify more. Determining when and where different isoforms are expressed, and finding any changes in expression in disease situations, is challenging. Use of single molecule fluorescent in situ hybridization (smFISH) techniques, which are being employed successfully in zebrafish (Soto et al., 2020; Stapel et al., 2016) will enable a detailed analysis of different splice forms and provide tissue‐specific cellular localization of the transcripts.

Another area requiring further investigation is the potential role of Adgrg6 as a mechano‐sensor, and whether mechanosensation is a key feature of Adgrg6 function in different tissues. It is interesting to speculate that Adgrg6‐mediated mechanosensing might have a similar role in the outgrowth and fusion of epithelial projections in the developing ear, as in skeletal development or cardiac trabeculation. Many similar genes and ECM molecules are expressed in all three organ systems and all are subject to mechanical forces. However, both heart and cartilage formation are thought to require the Adgrg6 NTF and do not appear to signal through the canonical cAMP pathway, whereas a role for the NTF alone has not been determined in the inner ear. Zebrafish are well suited to studying the mechanical contributions to tissue morphogenesis in vivo; a recent relevant example tackles the contribution of tension heterogeneity to cardiac trabeculation (Priya et al., 2020). The development and use of tools for live imaging and the measurement of mechanical forces in the zebrafish embryo are pushing boundaries in this important area (Serwane et al., 2017; Shah et al., 2019; Tlili et al., 2019; Tsai et al., 2020).

The pharmacological tools available for aGPCRs in general and Adgrg6 in particular are currently very limited. Hit compounds identified from existing or new chemical screens could be used to target different aspects of Adgrg6 biology using zebrafish in vivo assays, and could be developed into specific small molecule modulators of the receptor. Here, existing studies have already provided promising starting points, such as the gedunin class of molecules (Diamantopoulou et al., 2019; Stoveken et al., 2018). Virtual screening and in silico approaches used in conjunction with the existing and future receptor structures will also be important in identifying novel Adgrg6‐binding molecules.

Optimization of CRISPR/Cas9 technologies for zebrafish continues apace, with recent success in high‐efficiency targeted integration through homology‐directed repair using DNA or single‐stranded oligonucleotide templates (Prykhozhij et al., 2018; Wierson et al., 2020) or by direct base editing (see, for example, Rosello et al., 2021; Zhao, Shang, Ying, Cheng, & Zhou, 2020). Such approaches, as already exemplified by targeted disruption of the zebrafish Adgrg6 Ca^2+^‐binding site (Leon et al., 2020), can be used to introduce specific human disease mutations into the zebrafish gene or to create a humanized Adgrg6 gene in zebrafish, which will provide a valuable resource for ADGRG6 disease modeling and drug validation. Together, the multitude of approaches possible in the zebrafish offers great promise for furthering our understanding of the Adgrg6 signaling pathway, its roles in the developing embryo, and its significance for human disease.

## CONFLICT OF INTEREST

GRW is an employee and shareholder of Sosei Heptares. The other authors declare no competing interests.

## AUTHOR CONTRIBUTIONS

Sarah Baxendale, Anzar Asad, and Tanya T. Whitfield wrote the article, with contributions from Nahal O. Shahidan, and Giselle R. Wiggin. Anzar Asad, Sarah Baxendale, and Tanya T. Whitfield prepared the figures.

## Data Availability

Data sharing is not applicable to this article as no new data were created or analysed in this study.
